# Opportunities for Web-Based Indicators in Environmental Sciences

**DOI:** 10.1371/journal.pone.0042128

**Published:** 2012-08-15

**Authors:** Sergio Malcevschi, Agnese Marchini, Dario Savini, Tullio Facchinetti

**Affiliations:** 1 Dipartimento di Scienze della Terra e dell'Ambiente, University of Pavia, Pavia, Italy; 2 Dipartimento di Ingegneria Industriale e dell'Informazione, University of Pavia, Pavia, Italy; University of Maribor, Slovenia

## Abstract

This paper proposes a set of web-based indicators for quantifying and ranking the relevance of terms related to key-issues in Ecology and Sustainability Science. Search engines that operate in different contexts (e.g. global, social, scientific) are considered as web information carriers (WICs) and are able to analyse; (i) relevance on different levels: global web, individual/personal sphere, on-line news, and culture/science; (ii) time trends of relevance; (iii) relevance of keywords for environmental governance. For the purposes of this study, several indicators and specific indices (relational indices and dynamic indices) were applied to a test-set of 24 keywords. Outputs consistently show that traditional study topics in environmental sciences such as *water* and *air* have remained the most quantitatively relevant keywords, while interest in systemic issues (i.e. *ecosystem* and *landscape*) has grown over the last 20 years. Nowadays, the relevance of new concepts such as *resilience* and *ecosystem services* is increasing, but the actual ability of these concepts to influence environmental governance needs to be further studied and understood. The proposed approach, which is based on intuitive and easily replicable procedures, can support the decision-making processes related to environmental governance.

## Introduction

This paper introduces a method for deriving web-based indicators that represent the relevance (or popularity) of keywords related to key-issues of the ecological and sustainability sciences in different cultural contexts (scientific and everyday language). The multidisciplinary use of keywords will be taken into account by analyzing different types of web sources (search engines and their search options, citation databases). Furthermore, we propose the development of web profiles as a tool for supporting Strategic Environmental Assessment (SEA). More generally, we suggest that measures of relevance and variation in time of keywords might support the decision-making processes related to environmental policy, where different types of language (technical, interdisciplinary, everyday language) often meet.

New strategies for measuring and facilitating the integration of science and society are indicated amongst the “grand challenges” of earth system sciences for global sustainability by Reid et al [1]. Therefore, to gain a better understanding of current scientific progress and to manage the decision-making process in sustainable development more effectively, we can quantify the relevance and dynamics of the keywords related to Ecology used in different social contexts.

**Figure 1:**
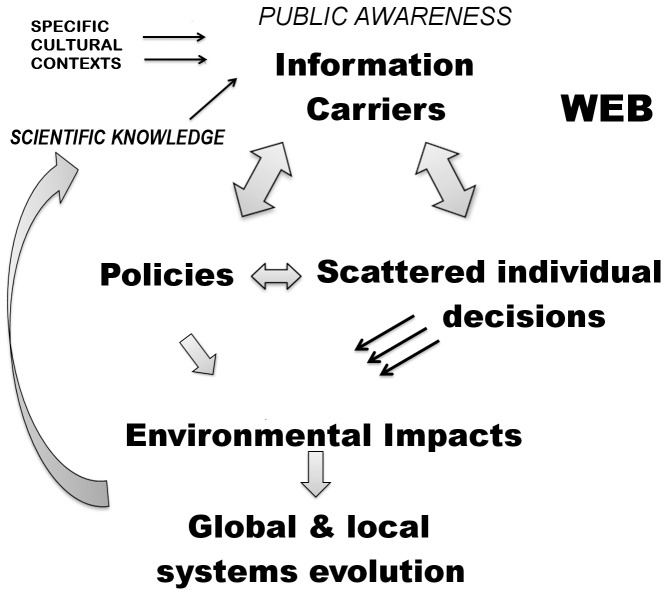
Conceptual model of the proposed approach. The meta-knowledge area, where scientific knowledge is combined with general knowledge (at the top) is the basis of decisions that produce impacts on the environment (in the centre), which in turn contribute to systems evolution (at the bottom). This process produces new scientific knowledge.

The current evolution of ecosystems is strongly influenced by human decisions (plans, programs, projects, actions, management), rather than by natural evolutionary processes. Such decisions are not only made by policy makers and administrators, but also by economic organizations and individual citizens (e.g. domestic economy, agriculture) (Fig. 1). A better understanding and management of the role these terms/concepts have in society might induce a proactive social reaction towards environmental conservation.

The most frequently used approaches to quantify scientific progress through keyword analysis have been bibliometric and scientometric, i.e. the analysis of citations and contents of scientific publications [2], [3], [4]. The analysis of citations has also been used to propose indicators of interdisciplinarity of scientific journals [5]; full articles, rather than their citations, have been used for mapping the structure of sciences [6].

A further analytical strategy is to explore the World Wide Web, which is currently the main system of communication. Society influences the web, and the needs of society are ever-changing. However, the web is also radically changing society and the way people (including scientists) access information and services, communicate, entertain themselves, express themselves as citizens or consumers, collaborate, work, educate and learn [7]–[10]. Search engines now represent the most widely used tool to access web content, and have a powerful role in shaping the web itself [11].

The information available on the web can be expressed by different descriptors and indicators such as documents, images, online news, either for general or for specific categories of users (e.g. scientists). Search engines –which we will henceforth refer to as “Web Information Carriers” (WICs)– can quantify the occurrence of single keywords either within subsets of the web (documents, news, etc.), or in the web as a whole.

Extracting information from WICs allows us to synthesize particular aspects of culture and its variability over time. On the other hand, the easy access to great amounts of data involves a high risk of finding junk data [12]. It is therefore important to perform research which aims to identify, set-up and validate the most effective indicators.

The use of WIC data for different purposes has significantly increased in recent years, thus broadening this research field [13], [14]. Web analytics applications have been widely used to derive statistics regarding the popularity of websites and specific webpage access. Such applications are particularly important in marketing research, e.g. to derive online brand positions [15]. WICs have also become important data mining instruments for biomedical applications [16], [17], [18].

The web search of scientific articles from the Web of Science databases has been used to compare different disciplines [19]; web archives from Google Scholar have been employed for the same purpose [20]. Geostatistical data from ISI - Web of Science, Scopus and Google Scholar have been compared by Hengl et al. [21]. Wikipedia has been used to produce an indicator of geopolitical instability [22]. The use of the Google Books tool has also been proposed as a useful integration of scientific databases where citations from most books and monographs are absent [23]. Google Books database has recently been used to analyse linguistic and cultural phenomena that were reflected in the English language between 1800 and 2000 in over 5 million digitized books [24].

The present article offers a new methodological approach that derives from these previous experiences. We strive to measure the relevance that ecological terms have on society in order to identify suitable keywords that could be used to effectively address a non-scientific community composed of stakeholders and citizens involved in participatory processes. Environmental sciences, like any other discipline, use keywords to synthesize and represent research areas: specific scientific terms restricted to specialists (i.e. *ecosystem services*); scientific terms that have started to permeate everyday language (i.e. *biodiversity*); terms used frequently in everyday language (i.e. *environment*). Different keywords have different effects on society and play different roles in influencing decisional processes.

In particular, we tested the hypothesis that WICs of differing specificity, from the most commonly used search engines (e.g. Google, Yahoo) to the most specific sectorial ones (e.g. New York Times-Archives, Web of Science), can provide consistent results by outlining emerging keywords as potential drivers for decision making in ecosystem management.

The present manuscript derives from preliminary investigations and applications of web profiling to SEA conducted by the first Author [25]–[29], one of the results of an Italian project named “Environmental & Cultural Web Profile” Project (ECWPP) [30].

## Methods

### Selection of keywords and web contexts

24 keywords were selected from those considered in ECWPP [30] (Table 1) with the aim of representing a variety of environmental aspects: primordial elements and living beings, systemic approaches, sustainable development aspects. Some personal/social sphere aspects were also included. The number and type of selected keywords allowed us to test the method and acknowledge its limitations and opportunities, with a view to further applications with more homogeneous and/or focussed sets of keywords.

**Table 1:** List of selected keywords for web profile identification.

Primordial entities and living beings	Sustainable development aspects
Water	Economy
Air	Society
Earth	Environmental Impact
Fire	Sustainable Development
Hydrogen	Energy
Oxygen	Photovoltaic
Biodiversity	Ecosystem Services
Grizzly	Resilience

Systemic approaches	Personal/social sphere aspects
Ecosystem	Culture
Territory	Life
Landscape	Sex
Environment	Love

Therefore, these 24 keywords are to be considered as a case study, rather than as targets of the present study.

Some of the selected keywords are restricted to specialist use, some are used in everyday language, and others belong to multiple semantic fields. In order to quantify and compare the popularity of the selected keywords under different conditions, they were analysed in different web contexts: scientific web, global web, social/personal web, web news. For each context, representative Web Information Carriers (WICs) were chosen.

### Selection of WICs, variables, indicators

In order to gather information about the relative importance of selected keywords at different levels, and to compare their dynamic behaviour, we considered various typologies of web-based indicators. There were two main technical types: (i) indicators of the relative importance of single keywords over the set of 24 keywords (relational indicators - *RI*), and (ii) indicators of emerging or stabilised temporal trends (dynamic indicators - *DI*). The spatial scale of indicators was heterogeneous: we selected indicators that act on a global level, on a national level, and in specific cultural contexts.

The selected web contexts, WICs, measured variables, proposed indicators and their typologies are listed in Table 2, and described in detail in the following sub-paragraphs. English was used as the reference language for all searches, as it often represents the default language in scientific and economic fields, as well as the most widely used language for information exchange. Searches were performed solely in English, in order to avoid overestimation of search results for terms that are used in several languages. Therefore, the “search the whole web” option, when available, was not considered. In some cases, the analysis of temporal trends is made possible by WICs that allow year-by-year searches.

**Table 2:** List of selected web contexts, and relative WICs used to perform searches.

Web domain	WICs	Measured variables	Indicators	Abbreviation	Type of indicator	
Scientific web	ISI - Web of Science data base	Number of articles containing a keyword	Percent of number of articles obtained in the recent 2 years (2009–2010) over the total number of articles obtained in 20 years (1991–2010) for a keyword	WOS.09–10%	R	G
			Emerging trends: percent variation of the period *t_(−1)_* (2009–2010) compared to the period *t_(−2)_* (2007–2008)	WOS.V1	D	G
			Stabilised trends: percent variation of the period *t_(−1)_* (2007–2010) compared to the period *t_(−2)_* (1991–1994)	WOS.V2	D	G
Global web	Google (english)	Number of web pages containing a keyword (median of replicates)	Percent of number of web pages obtained for a keyword over the total number of web pages obtained for all 24 keywords	WG.en%	R	G
	Yahoo! (english)	Number of web pages containing a keyword (median of replicates)	Percent of number of web pages obtained for a keyword over the total number of web pages obtained for all 24 keywords	WY.en%	R	G
	Google (english) & Yahoo! (english)		Arithmetic mean of Google and Yahoo! results (WG.en%, WY.en%) for a keyword	WGY.en%	R	G
Social web	Google Blog (english)	Number of web pages containing a keyword (median of replicates)	Percent of number of web pages obtained for a keyword over the total number of web pages obtained for all 24 keywords	WGB.en%	R	G-C
Web news	New York Times (Archives)	Number of articles containing a keyword	Percent of number of articles obtained in the recent 2 years (2009–2010) over the total number of articles obtained in 20 years (1991–2010) for a keyword	W.NYT.09–10%	R	G-N-C
			Emerging trends: percent variation of the period *t_(−1)_* (2009–2010) compared to the period *t_(−2)_* (2007–2008)	W.NYT.V1	D	G-N-C
			Stabilised trends: percent variation of the period *t_(−1)_* (2007–2010) compared to the period *t_(−2)_* (1991–1994)	W.NYT.V2	D	G-N-C
Individual web searches by users	Google Trends	Fixed-scale weighted index for a keyword in the USA	Emerging trends: percent variation of the period *t_(−1)_* (2009–2010) compared to the period *t_(−2)_* (2007–2008)	WGT.us.V1	D	N

The last two columns differentiate the following typologies of indicators: R = relational; D = dynamic; G = global level; N = national level; C = specific cultural contexts.

### Scientific web

In order to evaluate the presence of the selected keywords in the scientific area, searches were performed on the Thomson-Reuters ISI Web of Science database, i.e. “the world's leading citation database with multidisciplinary coverage of over 10,000 high-impact journals in the sciences, social sciences, and arts and humanities, as well as international proceedings coverage”. The preliminary survey on this WIC revealed a negligible variation of quantitative results within a time range of a few months. Therefore, searches on these databases were not replicated.

Since the Web of Science allows users to perform year-by-year searches, we collected yearly search results within the time range 1991–2010, in order to obtain temporal trends of the occurence of keywords in ISI publications. When comparing with other WICs, only recent search results were taken into account, namely the results achieved by searching the Web of Science archives in the years 2009 and 2010.

### Global web

Data harvesting was performed on the two search engines that currently dominate the web: Google and Yahoo! They provide the most comprehensive view of the contents of the World Wide Web by scanning images, videos, blogs, online news, books, scientific journals, social network updates, forum discussions.

A preliminary data collected from these two search engines had highlighted a fairly high coefficient of variation, with differences in orders of magnitude among quantitative results of searches of the same keyword performed at very short time intervals. In order to overcome this problem and to obtain reliable quantitative results, data were collected by periodically surveying two WICs (total occurences in English on Google and total occurences on Yahoo!) for one week (from 12/02/2011 to 18/02/2011), with 24 hourly replicates each day. For each replicate, the corresponding results page was analysed to extract the required information regarding the number of pages found by the WIC. In total, 168 replicates were performed for each keyword. Such a high number of replicates is recommended to reduce the dispersion of the distribution of quantitative results. The main location parameters of the data distribution (mean, median, mode) were then computed for each keyword in order to select the most suitable to use for the comparison of search result data. Finally, the percentage of occurrence of each keyword (over the total number of occurrences for all 24 keywords) was calculated and the total occurrences on Google and Yahoo! results were averaged. These data were then assumed to be indicators of the global web and used for comparisons with other WICs.

### Social web

The relevance of the selected keywords was also explored across a sphere reflecting the personal points of view of the Internet population by searching in a specific sub-set of the World Wide Web, i.e. the blog community. A blog (a blend of the term *web log*) is a type of website that is usually maintained by an individual who regularly publishes a commentary, descriptions of events, personal experiences and opinions, as well as links to other blogs or websites, pictures and videos. Most blogs work as personal online diaries, where people keep a running account of their personal lives. They also have a relevant role as an alternative source of news, as they allow personal points of view to be published that most politically-correct newspapers would not. Furthermore, blogs are more interactive than static websites, since they allow readers to leave comments, with or without moderation.

Therefore, blogs can be considered a good mirror of public opinion, reflecting personal points of view of the Internet population. A dedicated search of our 24 keywords was thus performed in the English-speaking blogosphere (collective community of all blogs), using the Google Blog search option.

Since preliminary surveys on this WIC highlighted relevant variations among quantitative results, replicated searches were performed, following the procedure described for the Global Web context.

### Web news

The WIC on the New York Times website was selected for the web news context.

The preliminary survey on this WIC had shown a fairly low coefficient of variation with time, so we collected results of single search events for every keyword. Since the New York Times search engine allows users to perform year-by-year searches, yearly search results were collected within the time range 1991–2010, in order to obtain temporal trends of keyword occurrence in the news. For the comparison with other WICs, only recent search results were taken into account, namely those from 2009 and 2010.

In the preliminary survey, the Google news service had also been considered as a potential WIC for the web news context, but it was not included in the final analysis because of the high variability of results.

### Individual searches by web users

Google Trends is one of the sub-sections of the Google search engine that “provides insights into broad search patterns”, i.e. estimates the volume of searches for a keyword by Google users at a given time and in a given place, starting from January 2004. Although the tool does not return real usage data, but estimates using weighted scale calculations, and declares that “several approximations are used when computing results”, Google Trends can be considered as a useful instrument to evaluate the information requests by web users and thus the actual level of public interest in a given term [31]. Results are plotted on a graph showing temporal trends of web traffic of the keyword, and it is possible for users to download a. csv file containing all the raw data.

Searches in Google Trends were performed by selecting the regional option “United States”, which avoids the overestimation of keywords that are used in several languages. On the other hand, it has the disadvantage of offering a partially representative view of the whole English-speaking community. For the comparison with other WICs, only recent search results were taken into account, namely those from 2009 and 2010 compared with those from 2007 and 2008.

### Meta-analysis: relational and dynamic indicators

The quantitative results for each keyword and each search variable were listed in worksheet tables.

Raw data were transformed into percentages (occurrence of a keyword over the total number of occurrences obtained by all 24 keywords in a WIC). We thus obtained the ranking of keywords within each WIC, which is useful for further comparisons between WICs.

Percentage occurrences of the 24 keywords obtained from WICs were processed in a multivariate analysis, aimed at finding similarities and dissimilarities between WICs and evaluating which keywords were mainly responsible for them. The input data for the analysis were: WG.en%, WY.en%, WGB.en% (median of replicated values), Web of Science, New York Times.

Similarity between pairs of WICs was computed using the Bray-Curtis index [32]. In order to identify groups of WICs characterized by similar rankings of the 24 keywords, a basic agglomerative hierarchical cluster algorithm was applied using the group average approach, which defines cluster proximity as the average pairwise proximity of all pairs of points from different clusters. This technique was selected as particularly recommended for a dendrogram plot based on the Bray-Curtis similarity index [33]. Keywords responsible for similarity within groups and dissimilarity between groups were identified by means of a similarity percentage (SIMPER) analysis, which examines the contribution of each variable (keywords) to the average resemblances/differences. Analyses were carried out using the software PRIMER version 6 [33]. We also compared selected pairs of WICs and quantified the different relevance of each keyword by means of a family of relational indices (*RI*):
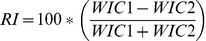
(1)where *WIC1* and *WIC2* stand for the percent occurrence of a keyword measured for two different WICs. Values of *RI* may range between +100, when a keyword only occurs in the *WIC1* results, and −100, when the keyword is completely absent in *WIC1* results. The considered pairs of WICs and relative type of *RI* values are shown in Table 3. The three considered *RI* were: an index of scientific relevance (*SRI*), an index of blogosphere relevance (*BRI*), and an index of news relevance (*NRI*). They quantify the importance of a given keyword in the scientific web, blogs or web news compared to the global web (averaged Google and Yahoo! percentage of the number of occurrences).

**Table 3:** Type of relational index (*RI*) and selected pairs of WICs considered for the comparison.

Type of *RI*	*WIC1*	*WIC2*	Object of the measurement by the *RI*	Type of indicator
*SRI*: Scientific impact	WOS.09–10%	WGY.en %	Relevance in the scientific community *versus* relevance in the global web	G
*BRI*: Blogosphere impact	WGB.en %	WGY.en %	Relevance in the blogosphere *versus* relevance in the global web	G
*NRI*: News impact	W.NYT.09–10%	WGY.en %	Relevance in the news *versus* relevance in the global web	G-N-C

See Table 2 for labels.

Finally, we explored temporal trends of relative variation for each single keyword, using yearly data collected from the following WICs: New York Times Archives, ISI Web of Science, and Google Trends. We distinguished between “emerging trends” (*V1*), i.e. those observed in the past 4 years (2007–2010), and “stabilised trends” (*V2*), i.e. those observed in the time range of two decades (1991–2010).

The evaluation of emerging *vs* stabilised trends was performed by means of a family of dynamic indices (*DI*), which aim to compare the percentage of the number of occurrences of a keyword in two different temporal phases: recent times (*t_(−1)_*) versus former times (*t_(−2)_*), thus allowing us to evaluate the type of temporal trend exhibited by a keyword. The proposed equation is similar to the one used for the family of *RI*:
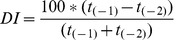
(2)where *t_(−1)_* and *t_(−2)_* account for the percentage of the number of occurrences of a given keyword in recent and former times, respectively. We provided two different interpretations of *t_(−1)_* and *t_(−2)_*, for emerging trends and stabilised trends (Table 4; see also Table 2). Since the Google Trends tool generates data starting from the year 2004, this WIC could only provide emerging trends.

**Table 4:** Type of dynamic index (*DI*) and relative WICs.

Type of *DI*	WICs	Total time range	*t_(−1)_* – recent times	*t_(−2)_* – former times
Emerging trend (*V1*): variations of a keyword occurrence in the past 4 years	WOS.V1; W.NYT.V1; WGT.us.V1	4 years; *t_(−1)_* and *t_(−2)_* last 2 years each	2009–2010	2007–2008
Stabilised trend (*V2*): variations of a keyword occurrence in the past 20 years	WOS.V2; W.NYT.V2	20 years; *t_(−1)_* and *t_(−2)_* last 4 years each	2007–2010	1991–1994

See Table 2 for labels. Different interpretations of the “recent times” and “former times” concepts offer an evaluation of emerging trends and stabilised trends of keywords occurrences.

The *DI* index may range between +100, for “brand new keywords”, which had only appeared in recent times, and −100, for “old-fashioned keywords”, which were only present in former times.

## Results

### Search results

Table 5 reports the results of data collection on the WICs that represent the current situation: Web of Science (occurrences in the years 2009–2010), global English-speaking web (averaged results of Google and Yahoo!) and New York Times (occurrences in the years 2009–2010).

**Table 5:** Results of data collection from selected WICs for the scientific web, global web, social web and web news contexts (see Table 2 for WIC labels).

Keywords	WOS.09–10%	W.GY.en%	W.GB.en%	W.NYT.09–10%
water	**17,5%**	8,1%	7,7%	7,3%
air	5,5%	9,2%	7,5%	8,2%
earth	2,0%	3,6%	3,5%	2,9%
fire	0,7%	5,4%	4,5%	5,8%
hydrogen	6,2%	0,2%	0,1%	0,2%
oxygen	6,5%	0,5%	0,3%	0,4%
ecosystem	1,2%	0,2%	0,1%	0,3%
territory	0,4%	1,1%	0,4%	2,6%
landscape	1,3%	1,4%	0,6%	2,2%
environment	8,1%	6,4%	3,3%	4,0%
economy	1,3%	3,8%	2,8%	**11,7%**
society	7,3%	5,1%	3,6%	5,5%
culture	5,8%	5,8%	3,4%	5,7%
life	11,9%	**18,7%**	**26,3%**	**20,6%**
sex	4,2%	6,5%	4,5%	3,7%
love	0,3%	**16,7%**	**25,7%**	**10,5%**
grizzly	0,0%	0,1%	0,1%	0,1%
environmental impact	0,2%	0,4%	0,1%	0,2%
sustainable development	0,3%	0,4%	0,1%	0,1%
energy	**17,3%**	6,0%	5,1%	7,6%
photovoltaic	0,5%	0,1%	0,0%	0,1%
biodiversity	1,0%	0,2%	0,0%	0,1%
ecosystem services	0,1%	0,1%	0,0%	0,0%
resilience	0,4%	0,1%	0,0%	0,30%

Raw data were transformed into percentage of occurrences of a given keyword over the total number of occurrences obtained from all the 24 keywords. Bold character style indicates the most popular keywords for each WIC; underlined style indicates the least popular keywords.

#### Scientific Web

The most successful keywords in scientific literature in the 2009–2010 biennium were *water* and *energy*. Other common keywords were *life*, *environment*, *society*, *oxygen* and *hydrogen* (Table 5). The least frequent terms were *sustainable development*, *environmental impact* and *ecosystem services*. The absolute least frequent term was *grizzly*, which appeared only about 700 times from 1991 to 2010 (i.e. about 35 times per year).

#### Global Web

The replicated searches performed on Google and Yahoo! for each keyword returned a set of results with high dispersion, confirming the observations made during the preliminary survey on these WICs. For example, the search of the keyword *economy* performed on Google returned a variable number of results within the range 304,000,000 to 923,000,000. Yet in Yahoo! the range was much wider: from 101,000,000 to 1,510,000,000. Data obtained by both search engines described strongly asymmetrical bimodal distributions skewed to the right. Only a small number of search results clustered in the proximity of the lower mode, which we considered less representative of the real number of pages found for the searched keyword, and more related to irregular behaviour of the search engines. Similar outputs were obtained for the other keywords, with Yahoo! usually providing a higher number of results than Google (two times higher on average). The only exception was for *sex*, which was most represented in Google. In all cases, Yahoo! provided a higher data dispersion than Google. For both Google and Yahoo!, we selected the median of the data distribution as a location parameter because it allows outliers to be removed due to irregular behaviour of search engines. Therefore, the median of temporal replicates was used to compare quantitative results between keywords. Despite quantitative differences, keyword ranking was very similar for the two search engines. The averaged percentage of occurrence of each keyword from Google and Yahoo! provided the ranking of terms for the global web context (English speaking) (Table 5). The top five keywords of the global web were: *life*, *love, air, water*, and *sex*, whereas the least successful keywords were: *grizzly, resilience, ecosystem services*, and *photovoltaic.*

#### Social Web

Data collected from Google Blog followed an irregular, multimodal distribution, although they had a smaller overall dispersion than data from Google and Yahoo!. The median was assumed as a suitable location parameter. Ranking highlights terms of social interest such as *life* and *love*, that were the most popular keywords in the blogosphere (Table 5). They occurred in three times as many pages as the following most successful terms, which are two archetypes of human cultural history: *water* and *air*.

Web News – The main terms in the personal sphere, *life* and *love*, and the main primordial elements, *air* and *water*, which had already emerged in the previous web contexts, were also identified as top keywords in the analysis of the New York Times archives (Table 5). A high ranking was also obtained by keywords related to society growth and development: *energy* and *economy*, the latter being much more successful in the New York Times news than in the rest of the web.

**Figure 2:**
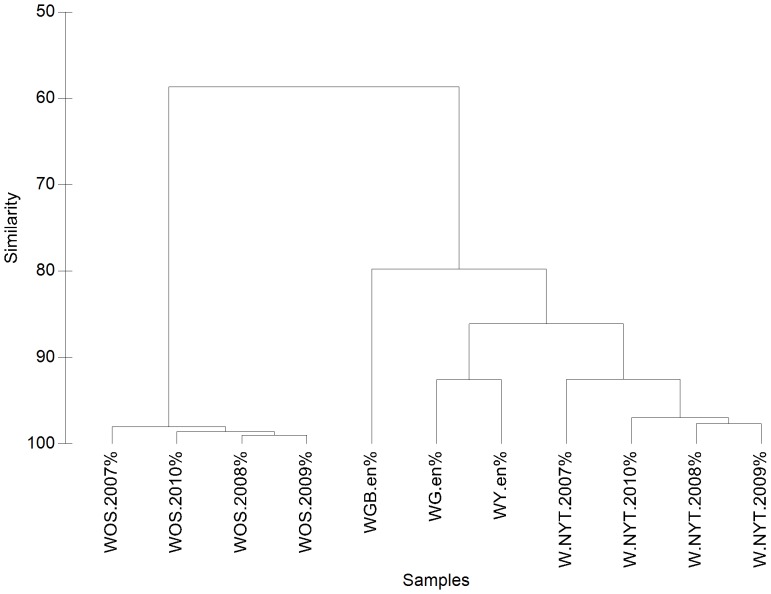
Group average dendrogram of WICs output; similarity is computed on the percent ranking values of the 24 selected keywords.

The results of the similarity analysis performed on the proposed WICs are synthesized in Fig. 2. The dendrogram shows that WICs are grouped in consistent clusters at high similarity levels (always >80%). The most clearly recognizable cluster represents the Web of Science results from 2007 to 2010. The web news WIC is very similar to the global web and the blogosphere.

SIMPER analysis identified the keywords that contributed the most to the observed dissimilarities between groups of WICs. The dissimilarity between scientific web and global web was mainly due to the terms: *photovoltaic*, *resilience*, *sustainable development*, *hydrogen*, *environmental impact*, *biodiversity*, *ecosystem*, *ecosystem services*, and *oxygen*, which were more relevant in the scientific web than in the global web, and to *life* and *love*, which were more relevant in the global web than in the scientific web. In other words, the scientific context gave more importance to specific ecological terms than the rest of the web, thus showing that for some keywords a gap exists between the scientific community and society as a whole.

### Relational and dynamic indicators

The proposed family of relational indices (*RI*) provided a quantification of the differences between pairs of web contexts (Table 6). The index *SRI* (WOS.09–10% *versus* W.GY.en%) highlighted that *hydrogen*, *oxygen*, *photovoltaic*, *ecosystem*, *biodiversity* and *resilience* were used a great deal more in the scientific publications than in the global web. The term with the lowest scientific relevance was *love*, which is a pure expression of personal human experience.

**Table 6:** Results of the relational indices (*RI*) and dynamic indices (*DI*) (See Tables 3 and 4 for labels and indices structure).

Keywords	SRI	BRI	NRI	WOS.V1	WOS.V2	W.NYT.V1	W.NYT.V2	WGT.us.V1
water	36,4	−2,6	−5,2	6,2	43,9	0,9	24,5	−0,3
air	−25,4	−10,2	−5,7	5,3	42,7	−1,5	13,1	1,1
earth	−29,2	−1,1	−10,3	3,7	39,2	−1,2	29,7	−8,1
fire	−76,4	−8,6	3,6	5,1	49,8	−1,1	16,5	−5
hydrogen	**93,7**	−16,1	−4,8	4,4	39,6	−5,9	28,7	−3,8
oxygen	**86,2**	−22,6	−5,3	4	31,3	−2,9	30,5	0,8
ecosystem	73,6	−17,5	23,1	9,8	73,9	13,2	53,7	1,9
territory	−46,5	−49,4	39,8	8,4	45,5	22	24,4	0,7
landscape	−3,5	−39,0	21,6	8,1	74,5	1,5	35,5	−6,6
environment	11,3	−31,8	−23,5	8,2	53,3	−2,5	31,9	−3,6
economy	−48,8	−15,5	**50,7**	10,4	38,9	19,6	29,4	−1
society	18,2	−16,5	4,1	12,5	75,4	2,7	13,3	−7,3
culture	0,3	−26,4	−1,0	5,7	23,8	3,4	38,5	−2,3
life	−22,0	**16,9**	4,8	7,9	55,1	2,4	22,1	−2,4
sex	−21,6	−17,9	−27,4	6,6	40,7	0,9	25,3	6,3
love	−95,9	**21,2**	−22,7	5,6	31,8	3,7	38,7	5
grizzly	−81,3	−30,5	−0,6	10,2	57,7	12,7	56,8	−2,4
environmental impact	−28,4	−70,7	−39,5	12	67,5	−2,8	36,8	−0,8
sustainable development	−25,9	−69,9	−73,5	15,5	73,6	8,7	57,5	−7,9
energy	48,7	−7,4	11,7	6,1	37,5	2,8	39,3	3
photovoltaic	**78,0**	−20,1	26,5	26,5	78,3	27,7	73,4	−2,4
biodiversity	70,2	−64,2	−27,7	12,3	90,8	4,4	49,3	1,4
ecosystem services	11,4	−57,2	−83,5	33,6	99,4	77,8	100	100
resilience	53,5	−37,0	**50,4**	20,3	85,3	17,4	52,9	11,2

The relative occurrence of most keywords was very similar in the blogs and in the global web, indeed the *BRI* index applied to WGB.en% *versus* W.GY.en% produced positive values close to 0. The most relevant negative values were for *sustainable development* and *environmental impact,* meaning that these themes were generally disregarded by bloggers.

The New York Times archives was also similar to the global web: the *NRI* index (NYT.09–10% *versus* W.GY.en%) produced results that were fairly close to 0 for many keywords. Points of divergence could be explained by the specific cultural area addressed by the newspaper, as in the case of the keyword *economy*.

The year-by-year search of the 24 keywords on the Web of Science in the time range 1990–2010 showed a very remarkable increase with time in the number of search results. All terms, with the exception of *grizzly*, reached their maximum occurrence in ISI publications in 2009 or 2010. This can be explained by the continuous increase in the number of indexed journals, which is partially related to the establishment of their online versions. But apart from the “physiological” growth in number of occurrences, there were noteworthy differences between keywords in terms of their growth trends.

**Figure 3:**
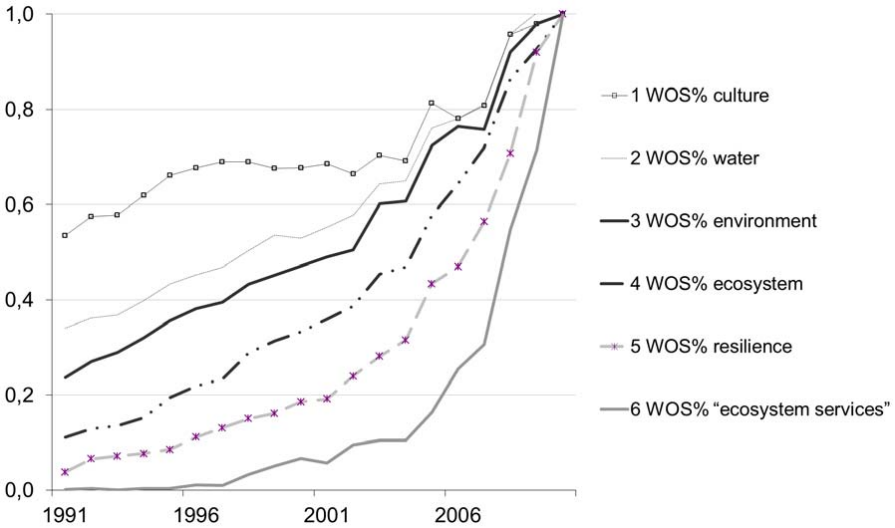
Types of temporal trends obtained from the year-by-year searches in the Web of Science databases: results for 6 selected keywords. Data are expressed as ratio over the maximum value, achieved in the year 2010.

Successful keywords, such as *water*, *culture*, *hydrogen*, *oxygen*, *air* and *energy*, showed moderate growth, whereas some less popular keywords, such as *ecosystem services* and *resilience*, showed a more marked growth trend (Fig. 3). There were also several terms with an intermediate behaviour, e.g. *environment* and *ecosystem*.

The keyword *culture* displayed the smallest relative growth: it had an average of about 17,000 occurrences in the early 1990s, and about 26,000 occurrences in the late 2000s, showing an increase of “only” about 150% (stabilised trend: *V2* = 23.8). On the contrary, although *ecosystem services* displayed a very small number of occurrences compared to most of the other keywords (from a few units in the 1990s to a few hundreds in the 2000s), it exhibited the most extraordinary relative growth, and the highest values of the *DI* both for emerging and stabilised trends (*V1* = 33.6; *V2* = 99.4) (Table 6).

The analysis of dynamic variations of the 24 keywords in the New York Times archives also returned a variety of temporal trends. The relative importance of the most popular keywords, *life*, *love*, *air*, *water*, *energy*, *economy*, remained constantly high in the two considered decades. Very different behaviour was observed for another set of keywords, which, despite being scarcely relevant in terms of relative number of occurrences, exhibited an exponential increase during recent years. It is interesting to note that these keywords all belong to the ecology/environment subject area, with particular reference to the systemic approach: *ecosystem*, *biodiversity*, *environmental impact*, *sustainable development*, *resilience*, *photovoltaic* and *ecosystem services*. The last keyword in this list is the least important, with only 56 total occurrences in 20 years of New York Times archives, but it is the keyword with the most noteworthy increase, as proved by the high values obtained with the *DI* for both emerging and stabilised trends (*V1* = 77.8; *V2* = 100).

The temporal dynamics provided by the Google Trends tool were irregular and governed by sudden changes. The following keywords received constantly high attention from American web users within the considered time range: *earth*, *life, energy* and *resilience*. Conversely, the keywords that received low interest from American web users were: *culture*, *society*, *environment*, *sustainable development* and *biodiversity*. *Ecosystem services* presented the most irregular behaviour: up to 2009 it had almost been ignored by Google users, then in 2010, there was a sudden explosion of interest.

In order to summarise the results of the most meaningful web-profile indicators on the basis of our study objectives (WOS.09–10%; WOS.V1; WOS.V2; WGY.en%; WGB.en%; WGT.us.V1 – see Table 2 for labels), we plotted radar graphs for 7 selected keywords: *water*, *life*, *love*, *ecosystem services*, *resilience*, *ecosystem*, *environment* (Fig. 3). These graphs highlighted differences in popularity between:

- representative keywords from different subject contexts (see Table 1): primordial entities (*water*), personal sphere (*life*, *love*), sustainable development (*ecosystem services*, *resilience*), and systemic approaches (*ecosystem*, *environment*);
- specific scientific terms (i.e. *ecosystem services*) compared to both scientific terms that have started to permeate everyday language (i.e. *ecosystem*), and scientific terms that are used in everyday language (i.e. *environment*).

Furthermore, the radar graphs displayed the results according to four different interpretation criteria: (c1) relevance in the scientific context: WOS.09–10%, WOS.V1, WOS.V2; (c2) current relevance in the world: WOS.09–10%, WGY.en%, WGB.en%; (c3) relevance in the personal sphere: WGB.en%, WGT.us.V1; (c4) emerging and stabilised trends of dynamic variation: WOS.V1, WOS.V2, WGT.us.V1. This analysis did not consider more specific web contexts, such as the web news expressed by the New York Times archives.

**Figure 4:**
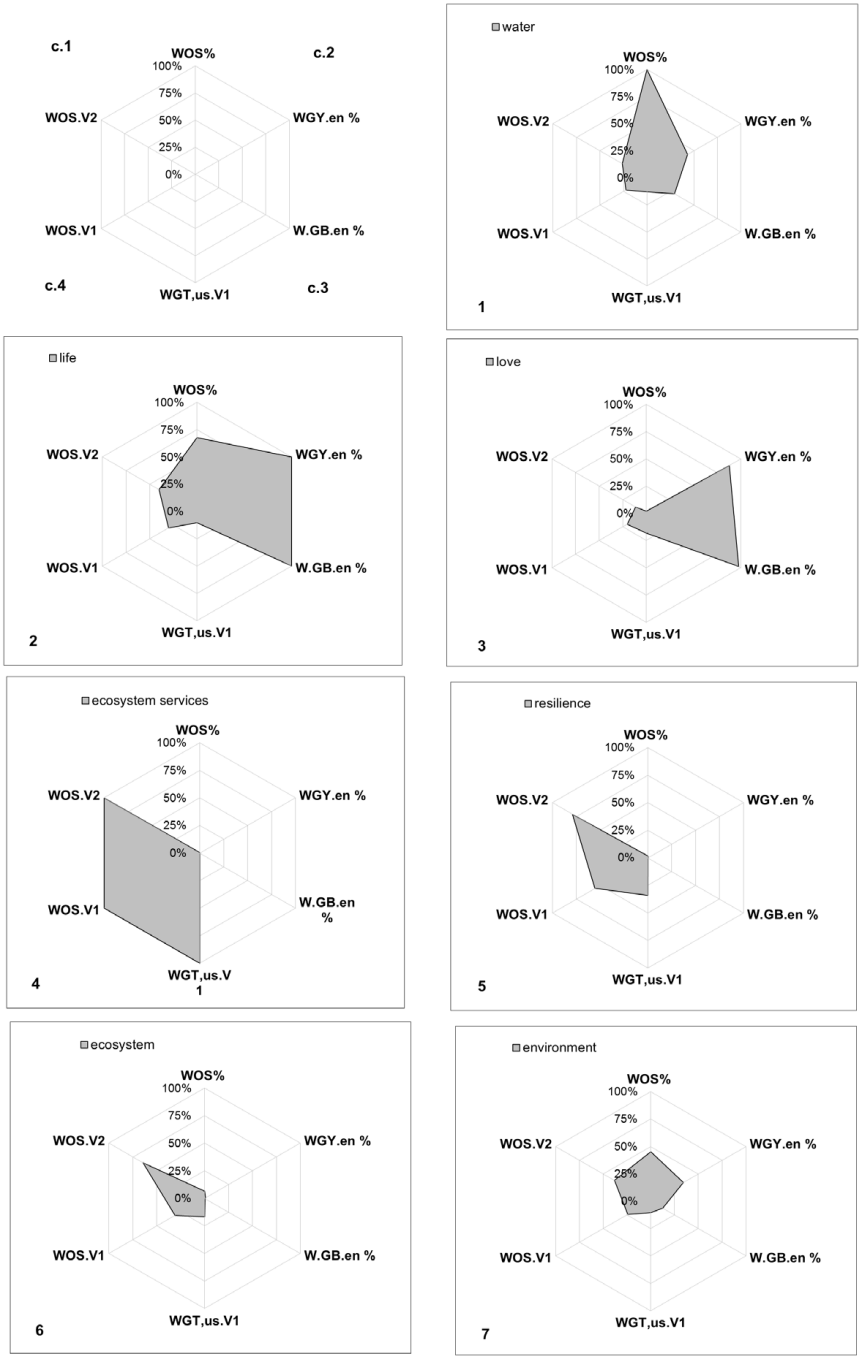
Radar graphs of 7 selected keywords according to the following web-profile indicators: WOS.09–10%; WOS.V1; WOS.V2; WGY.en%; WGB.en%; WGT.us.V1 (see **Table 2** for labels). The interpretation criteria are: (c1) relevance in the scientific context; (c2) current relevance in the world; (c3) relevance in the individual sphere; (c4) emerging and stabilised trends of dynamic variation. For each WIC, relative percent values over the total of 24 keywords are reported.

Figure 4 demonstrates that *life* was the most widespread keyword, due to its high relevance in both scientific and general/social contexts (c1, c2, c3). There was also a moderate increase in its relevance (c4) in the Web of Science databases. *Water* received the most attention in the scientific context (c1), and high levels of attention in the other web contexts (c2, c3), but its relevance did not increase as significantly over time (c4). *Love* and *ecosystem services* were poles apart: the former dominated the personal sphere (c3) but increased negligibly over time (c4), whereas the latter was very unpopular in general/social contexts (c2, c3), but was the most relevant new keyword (c4). *Resilience* and *ecosystem* behaved in a similar way to *ecosystem services*. The web profile of the keyword *environment,* however, was midway between these two extremes.

## Discussion

### Keyword profiles in web contexts

The web profiles of the 24 selected keywords, which result from the proposed web-based indicators, highlight differences between the considered web contexts. Each individual keyword web profile is only meaningful when compared to other keyword web profiles, either from the same subject context, or from different contexts. The results of the proposed methodology, therefore, strongly depend on the set of reference keywords, which should be carefully selected for the purposes of the investigation as they define the contexts within which results are interpreted.

In our analysis, *water* was the most relevant keyword in the scientific context, but it also ranked highly in other cultural contexts. Water has indeed been one of the main subjects of human knowledge and research since primordial accounts of human intellect dating back 2,500 years (e.g. Thales of Miletus). Knowledge on water is not limited to the ecological field, it is also relevant in a large number of physical, humanistic, and practical fields of study. Similar web profiles, albeit with fewer occurrences, were produced for *air* and *fire*. Surprisingly, they proved to be more relevant in the global web, social web and web news contexts than in the scientific web context. *Energy*, like *water*, was another keyword that received similarly high attention across all the web contexts. These popular keywords also shared similar dynamic behaviour: their relevance only moderately increased over time, making them “evergreens” in both the scientific and the public interest contexts.

A very different pattern was exhibited by *ecosystem services*, *resilience*, *environmental impact*, *ecosystem*, and *sustainable development*. Overall, they were much less relevant than the most popular keywords such as *water* (in terms of number of occurrences), but they increased more significantly in the 2000s both in the scientific and in the public interest contexts. These results demonstrate that there is an increase in public awareness and concern about systemic aspects and sustainability. The most relevant example is the keyword *ecosystem services*, which was by far the least popular keyword, ranking 24^th^ in web news and blogosphere, and 23^rd^ in the global web and scientific web. However, it was the most interesting “brand new keyword”, expressing the concept of “novelty” to the highest degree for all dynamic variation indicators. It showed exponential growth not only in the scientific context (Web of Science databases), but also in the social contexts, expressed by the New York Times Archives and Google Trends (USA users).

Some keywords had average results, for example *environment*, *earth*, *biodiversity* and *landscape*. Their occurrences on the web and trends in increased usage were moderately high, but they were neither highly ranked, nor brand new keywords in science, news and society. *Environment* is probably the keyword which best synthesised all the different societal contexts under examination. The challenge for the scientific community is to keep the level of public concern high about these keywords, thereby preventing them from becoming meaningless buzzwords.

Most of the keywords we selected were related to technical-scientific aspects of ecological sciences and sustainability, but the few keywords that were related to the personal sphere, i.e. *life* and *love*, were found to be the most popular in the global web, blogosphere and web news. In particular, *life* obtained the highest ranking out of all the considered web indicators, whereas the high level of attention observed for *love* in the global web, social web and web news was not confirmed in the scientific web, where it had very low relevance. *Love* was the only keyword that specifically addressed the non-scientific contexts, as it is closely linked to personal epistemological beliefs.

Fazey [34] suggested that the beliefs people hold about the nature of knowledge and how something is known might have profound implications on the way individuals relate to each other and the world, such as how people understand complex social-ecological systems. As Shields et al. [35] pointed out, science is effective when it is presented in a manner that is meaningful to the audience, and also represented in the context of their values and objectives, so overlaps between policy, science, and public values and objectives cannot be ignored. Therefore, it is a stimulating challenge for natural scientists to extend the boundaries of rigorous quantitative inquiry to social sciences and humanities, and to include relevant keywords in communication strategies, in order to develop a continuous dialogue and cooperation with social scientists, policy makers and citizens. In the near future, sustainability sciences might need to be reshaped and former certainties re-evaluated in the framework of innovative and multidisciplinary research approaches such as metaknowledge [36] and culturomics [24].

### WICs as indicators for ecological and sustainability sciences

In recent years there has been growing awareness within the scientific community of the relevance of communication and public participation in environmental management issues, in order to achieve credibility and legitimacy in society [1], [37]. Transdisciplinary approaches that can integrate social, economic and physical systems have been increasingly recommended to manage the complex issues involved in sustainability sciences [38], [39]. In order to achieve this objective, it is first necessary to recognise and acknowledge different knowledge claims on scale and governance, before they can be reflected upon and discussed in transdisciplinary arenas [40].

Our methodological proposal aims to meet the demand for innovative tools to identify knowledge patterns within the increasingly complex interaction between cultures, disciplines and systems involved in the environmental management process. The proposed web-based indicators might act as a bridge between physical and social analyses.

Web-profile analysis might help ecologists and sustainability scientists to face current challenges that require integrated approaches, such as the biodiversity conflict [41], which is embedded in ecological, economic and social contexts. For example, one of the challenges that the field of biodiversity conservation faces is the fact that most financed conservation actions have been obtained to protect “charismatic” taxonomic units at the expense of other taxa, which are less popular or even lesser known, whose role in trophic webs and ecosystem functioning would possibly be more relevant [42], [43], [44]. In this field, the web-profile approach could strengthen other approaches that are based on traditional bibliometric analyses [45]. SEA (Strategic Environmental Assessment) is another rapidly expanding field where environment and society meet and where knowledge brokerage is promoted, which poses the problem of identifying appropriate effective keywords for facilitating knowledge exchange and transfer as part of assessment processes [46]. In this framework, WICs might act as indicators of specific local conditions, as well as indicators of complex relationships among natural (ecological), economic and human (socio-political-institutional) subsystems, along spatial and temporal scales, whose importance has been highlighted by Ostendorf [47].

It has been observed that in places with high living standards, the population is more aware of environmental impairment and therefore more inclined to invest in environmental protection [48]. Consequently, web indicators on a local scale (national or sub-national) should be considered in order to understand local dynamics that might influence the selection process of environmental protection actions. For example, online news website archives might provide an insight into different countries or even towns within countries.

Therefore, the web-profile approach offers a common framework within which keywords of different epistemological nature and in different cultural contexts can be analysed. This approach can also deal with problems of scale, a crucial topic in the fields of public administration, political sciences, and environmental sciences [40], [49].

### Strengths, weaknesses, opportunities and threats

The development of effective indicators and indices, i.e. representative of complex systems, easily communicable and robust from an analytical and statistical standpoint, is not a trivial task. Our approach involving web-based indicators satisfies some of the “good indicator” requirements [50], but more research is needed to improve its reliability and relevance, and to define procedural standards for routine applications. In this framework, strengths, weaknesses, opportunities and threats of the current status of web-based indicators can be identified, according to the SWOT analysis approach [51].

One strong point of the proposed web-based approach is its ability to synthesise the large amounts of information contained on the web. By quantifying total search results, we avoided considering only the small subsets of information that appear on the first few results pages of search engines. Data collected from single WICs, which represent specific web contexts and related cultural worlds, summarise the relative importance and behaviour in the time of selected keywords. Furthermore, the pervasiveness of the web in today's society means that a web-based approach is widely comprehensible by the public, press, and policy makers. Data collection is highly feasible as the only requirements are a computer and an Internet connection, and it is much faster than any other manual literature survey.

However, the reliability of collected data is questionable, for at least three reasons. Firstly, collected data reflect specific aspects of the selected linguistic area, in this case the English-speaking web, and we cannot exclude the fact that contrasting results might be obtained by exploring different linguistic areas. Secondly, the behaviour of search engines might respond to local Internet policies, such as website filtering, thus data collection performed in different countries might return different results. Thirdly, collected data depend on the functioning of web search engines, but the algorithms that govern search engines are industrial secrets and are continuously modified and upgraded. Therefore, the main threats to the proposed methodology are that the technicalities of using WICs need further validation, and that WICs behave and evolve unpredictably. Moreover, the World Wide Web is an extremely dynamic system, and its relationships with society are complex and rapidly evolving [52]. As a result, the relevance of using quantitative indicators to describe the evolution of information on the web should be confirmed by tests under different conditions. The conceptual model that the approach is based on is simple and straightforward. However, web-based indicators are still far removed from ordinary fields of knowledge and research, and there are no standardised procedures. In our opinion, this aspect represents the weakest point of the proposed approach, as with most novel approaches in the field of sustainability. Thus, further issues will have to be tackled in order to receive acknowledgement from the scientific community.

Other features of “good indicators” [50], however, might represent important opportunities for future research development and improvement. The option of identifying a limited set of standardised reference keywords to be used for comparisons and specific analyses is highly stimulating and challenging. We believe that some of the keywords that we propose in this paper could actually be considered for this purpose, for example *water* or *environment*.

The identification of the most relevant fields for practical implementation of web-based indicators, their actual application to decisional processes, and the *post-hoc* evaluation of their effectiveness in driving management actions are the most interesting opportunities for future research.

## Conclusions

This work presents a method based on the World Wide Web to analyse relationships between society and scientific progress, and to integrate them into SEA (Strategic Environmental Assessment) procedures. In particular, we propose a combination of several web-based indicators to construct web profiles of single keywords. Web profiles are then suggested as potential models for the interpretation of information within different cultural contexts. Our results clearly show an increase in society's interest in relatively new ecological terms of the sustainable and developmental sphere (i.e. ecosystem services, resilience). In the context of territory governance, web-based indicators could be used to improve public/administrator awareness of terms specific to ecological sciences and sustainable development.

The proposed approach requires further improvements to better identify the functioning of the proposed indicators. We outline the following points for potential improvement:

- Other web contexts and websites could be considered as suitable WICs to perform data mining, to produce additional data and indicators, and thus to define more exhaustive web profiles. For example useful information could be gathered from: wider scientific databases (Google Scholar), digitised books (Google Books), images (Google Images, Yahoo! Images), satellite images (Google Earth), videos (Google Video, Yahoo! Video, You Tube), online news on the whole web (Google news), social networks (Google Realtime, Google Discussions, Facebook, Twitter).
- Improved indices to measure scientific specificity of keywords with respect to other web contexts and collective psyche could be developed.
- Analyses of co-occurrence of keywords could be carried out, such as those already performed with traditional blibliometric [4] or web-based [15] approaches.
- Web profiles could be derived from specific geographical areas by setting the language options of search engines and using local newspapers websites.
- Different algorithms could be considered to establish indicators, for example relative relevance of keywords could be computed considering different reference conditions.
- Indicators and indices could be developed as a support of local decision-making processes in the framework of SEA.
- Web profiles could be used as indicators to monitor various broad areas of expertise (aspects of society, economy, local administration, events), as support for decisional processes.
- WICs could be used as instruments in the research field of metaknowledge [36], which is located midway between science and culture.

## References

[1] Reid WV, Chen D, Goldfarb L, Hackmann H, Lee YT, et al (2010) Earth system science for global sustainability: grand challenges. Science 330: 916–917. 21071651 10.1126/science.1196263

[2] Hood WW, Wilson CS (2001) The literature of bibliometrics, scientometrics, and informetrics. Scientometrics 52: 291–314.

[3] Moed HF, Glänzel W, Schmoch U (2004) Handbook of Quantitative Science and Technology Research, 2004 Kluwer Academic Publishers. Printed in the Netherlands 800.

[4] Nobis M, Wohlgemuth T (2004) Trend words in ecological core journals over the last 25 years (1978–2002). Oikos 106: 411–421.

[5] Leydesdorff L (2007) Betweenness centrality as an indicator of the interdisciplinarity of scientific journals. J Am Soc Inf Sci Tec 58: 1303–1319.

[6] Bollen J, de Sompel HV (2006) Mapping the structure of science through usage. Scientometrics 69: 227–258.

[7] Slevin J (2000) The internet and society. Oxford: Wiley-Blackwell. 266 p.

[8] Yang G (2003) The co-evolution of the internet and civil society in China. Asian Surv 43: 405–422.

[9] Berners-Lee T, Hall W, Hendler J, Jweitzner DJ (2006) Creating a Science of the Web. Science 313: 769–771. 16902115 10.1126/science.1126902

[10] Cortese J (2007) Internet learning and the building of knowledge. Amherst, New York: Cambria Press.183p.

[11] Introna LD, Nissenbaum H (2000) Shaping the web: why the politics of search engines matters. Inform Soc 16: 169–185.

[12] Schwarz CJ (2007) Computer-aided statistical instruction - Multi-mediocre techno-trash? Int Stat Rev 75: 348–354.

[13] Cooley R, Mobasher B, Srivastava J (1997) Web mining: information and pattern discovery on the World Wide Web. 9th International Conference on Tools with Artificial Intelligence (ICTAI '97) 558.

[14] Fergus R, Perona P, Zisserman A (2004) A visual category filter for Google images. Proc ECCV 3021: 242–256.

[15] Aggarwal P, Vaidyanathan R, Venkatesh A (2009) Using lexical semantic analysis to derive online brand positions: an application to retail marketing research. J Retailing 8: 145–158.

[16] Gaikwad J., Khanna V, Vemulpad S, Jamie J, Kohen J, et al. (2008). CMKb: a web-based prototype for integrating Australian Aboriginal customary medicinal plant knowledge. Available: http://www.biomedcentral.com/1471-2105/9/S12/S25 via the Internet. Accessed 04/12/2011. 10.1186/1471-2105-9-S12-S25 PMC2638165 19091025

[17] Seifter A, Schwarzwalder A, Geis K, Aucott J (2010) The utility of “Google Trends” for epidemiological research: Lyme disease as an example. Geospatial Health 4: 135–137. 20503183 10.4081/gh.2010.195

[18] Ortiz JR, Zhou H, Shay DK, Neuzil KM, Fowlkes AL, et al (2011) Monitoring Influenza Activity in the United States: A Comparison of Traditional Surveillance Systems with Google Flu Trends. PLoS ONE 6 4:e18687 doi:10.1371/journal.pone.0018687. 21556151 10.1371/journal.pone.0018687 PMC3083406

[19] Vaughan L, Shaw D (2005) Web citation data for impact assessment: A comparison of four science disciplines. J Am Soc Inf Sci Tec 56: 1075–1087.

[20] Noruzi A (2005) Google Scholar: The new generation of citation indexes. Libri 55: 170–180.

[21] Hengl T, Minasny B, Gould M (2009) A geostatistical analysis of geostatistics. Scientometrics 80: 491–514.

[22] Apic G, Betts MJ, Russell RB (2011) Content Disputes in Wikipedia Reflect Geopolitical Instability. PLoS ONE 6 6:e20902 doi:10.1371/journal.pone.0020902. 21731630 10.1371/journal.pone.0020902 PMC3120813

[23] Kousha K, Thelwall M (2009) Google book search: citation analysis for social science and the humanities. J Am Soc Inf Sci Tec 60: 1537–1549.

[24] Michel JB, Shen YK, Aiden AP, Veres A, Gray MK, et al (2011) Quantitative analysis of culture using millions of digitized books. Science 331: 176–182. 21163965 10.1126/science.1199644 PMC3279742

[25] Malcevschi S (2005) Analisi semantica mediante Web come strumento per la definizione delle priorità di interesse in campo ambientale. Valutazione Ambientale 8: 11–14.

[26] Malcevschi S (2005) L'ambiente urbano: aspetti emergenti da un'analisi semantica mediante Web. Valutazione Ambientale 8: 47–51.

[27] Malcevschi S (2008) Territorio, Paesaggio. Trend on-line delle attenzioni. Valutazione Ambientale 14: 85–87.

[28] Malcevschi S (2009) Due indicatori preliminari per l'analisi dei flussi informativi in campo ambientale sul Web: l'indice WP% di rilevanza relativa e l'indice NOLT di tendenza recente. Valutazione Ambientale 16: 87–91.

[29] Malcevschi S (2010) Google-Images descrive l'ambiente? Valutazione Ambientale 17: 105–109.

[30] Malcevschi S (2010) “Environmental & Cultural Web Profiles” Project (ECWPP). Available: http://www.webprofileproject.eu/ via the Internet. Accessed 11 December 2011.

[31] Choi H, Varian H (2009) Predicting the present with Google Trends, Google Inc. Draft Date April 10, 2009. Available: http://googleresearch.blogspot.com/2009/04/predicting-present-with-google-trends.html#/2009/04/predicting-present-with-google-trends.html. Accessed 13 December 2011.

[32] Bray JR, Curtis JT (1957) An ordination of the upland forest communities of Southern Wisconsin. Ecol Monogr 46: 327–354.

[33] Clarke KR, Gorley RN (2006) PRIMER v6: user manual/tutorial. PRIMER-E, Plymouth.

[34] Fazey I (2010) Resilience and higher order thinking. Ecol Soc 15 3:9 Available: http://www.ecologyandsociety.org/vol15/iss3/art9. Accessed 13 December 2011.

[35] Shields DJ, Šolar SV, Martin WE (2002) The role of values and objectives in communicating indicators of sustainability. Ecol Indic 2: 149–160.

[36] Evans JA, Foster JG (2011) Metaknowledge. Science 331: 721–725. 21311014 10.1126/science.1201765

[37] Rametsteiner E, Pülzl H, Alkan-Olsson J, Frederiksen P (2011) Sustainability indicator development—Science or political negotiation? Ecol Indic 11: 61–70.

[38] Kemp-Benedict EJ, Bharwani S, Fischer MD (2010) Using Matching Methods to Link Social and Physical Analyses for Sustainability Planning. Ecol Soc 15 3:4 Available: http://www.ecologyandsociety.org/vol15/iss3/art4/. Accessed 13 December 2011.

[39] Perz SG, Brilhante S, Brown IF, Michaelsen AC, Mendoza E, et al (2010) Crossing boundaries for environmental science and management: Combining interdisciplinary, interorganizational and international collaboration. Environ Conserv 37: 419–431.

[40] Buizer M, Arts B, Kok K (2011) Governance, scale, and the environment: the importance of recognizing knowledge claims in transdisciplinary arenas. Ecol Soc 16(1), 21. Available: http://www.ecologyandsociety.org/vol16/iss1/art21/. Accessed 13 December 2011.

[41] White RM, Fischer A, Marshall K, Travis JMJ, Webb TJ, et al (2009) Developing an integrated conceptual framework to understand biodiversity conflicts. Land Use Policy 26: 242–253.

[42] Andelman SJ, Fagan WF (2000) Umbrellas and flagships: efficient conservation surrogates or expensive mistakes? Proceedings of the National Academy of Sciences USA 97: 5954–5959. 10.1073/pnas.100126797 PMC18540 10811901

[43] Entwistle A, Dunstone N (2000) Priorities for the Conservation of Mammalian Diversity: Has the Panda Had its Day? Cambridge University Press, Cambridge.

[44] Sergio F, Newton I, Marchesi L, Pedrini P (2006) Ecologically justified charisma: preservation of top predators delivers biodiversity conservation. J Appl Ecol 43: 1049–1055.

[45] Caliman A, Pires AF, Esteves FA, Bozelli RL, Farjalla VF (2009) The prominence of and biases in biodiversity and ecosystem functioning research. Biodivers Conserv 19: 651–664.

[46] Sheate WR, Partidário MR (2010) Strategic approaches and assessment techniques-Potential for knowledge brokerage towards sustainability. Environ Impact Asses 30: 278–288.

[47] Ostendorf B (2011) Overview: Spatial information and indicators for sustainable management of natural resources. Ecol Indic 11: 97–102.

[48] Arrow K, Bolin B, Costanza R, Dasgupta P, Folke C, et al (1995) Economic growth, carrying capacity, and the environment. Science 268: 520–521. 17756719 10.1126/science.268.5210.520

[49] Termeer CJAM, Dewulf A, van Lieshout M (2010) Disentangling Scale Approaches in Governance Research: Comparing Monocentric, Multilevel, and Adaptive Governance. Ecol Soc 15(4), 29. Available: http://www.ecologyandsociety.org/vol15/iss4/art29/ via the internet. Accessed 13/12/2011.

[50] Borja Á, Dauer DM (2008) Assessing the environmental quality status in estuarine and coastal systems: Comparing methodologies and indices. Ecol Indic 8: 331–337.

[51] Learned EP, Christensen CR, Andrews KE, Guth WD (1965) Business Policy: text and cases. Irwin, Homewood.

[52] Fuchs C (2008) Internet and society: social theory in the information age. Routledge: New York.

